# Genetic diversity of *Prosopis juliflora* in the state of Qatar and its valuable use against postharvest pathogen of mango fruits

**DOI:** 10.1038/s41598-022-14871-x

**Published:** 2022-06-30

**Authors:** Iman Saleh, Talaat Ahmed, Ream Halboosi, Mohammed Abu-Dieyeh

**Affiliations:** 1 Biological Science Program, Department of Biological and Environmental Sciences, College of Art and Science, Qatar University, P.O. Box 2713, Doha, Qatar; 2 Environmental Science Centre, Qatar University, P.O. Box 2713, Doha, Qatar

**Keywords:** Environmental impact, Biodiversity, Natural variation in plants, Plant biotechnology

## Abstract

Mango (*Mangifera indica*) is the second most internationally traded tropical fruit in the world. The fruit has high nutritional value. Its susceptibility to postharvest diseases and chill injuries increases its storage cost and put stress on exploring natural products that can increase its shelf-life. Our team has previously described *Prosopis juliflora* water-soluble leaf ethanolic (PJ-WS-LE) extract with fungicidal effectiveness against spoiling fungi. The present study explores *P. juliflora* genetic diversity in the state of Qatar and the antifungal effectiveness of the leaf extract of plants collected from different locations. The study also evaluates PJ-WS-LE extract efficacy against *Alternaria. alternata* and *Colletotrichum gloeosporioides* inoculated in mango samples and the power of the extract as coating material. *P. juliflora* samples collected from six different locations showed genetic and antimicrobial effectiveness similarities. They showed also similarity to the sequence representing *P. juliflora* 18S ribosomal RNA partial sequence, accession number JX139107.1 originated from India. PJ-WS-LE extract (8 mg/ml) has 80% efficacy in controlling *A. alternata* in mango and it lowers *C. gloeosporioides* disease severity by 53.4%. PJ-WS-LE extract (8 mg/ml) embedded in 1% chitosan maintained mango quality for 5 weeks. In vivo results of PJ-WS-LE extract highlights the potentials of the extract as chemical fungicides replacement.

## Introduction

*Prosopis* (mesquite; Fabaceae) is found in hot, dry and semi-arid areas around the world, either as native species or as introduced ones^1^. *Prosopis* is known as major invasive species in many countries^2^. *Prosopis juliflora* (Mimosaceae), the focus of this study, is a small tree, native to Mexico, South America and Caribbean, it is an increasingly spreading invasive species in Asia, Austria and in other places around the world including Qatar^3^. When considering the exploration of *P. juliflora* as source of biological controllers, it is important to learn first about its biodiversity. When introduced to the country, the tree may originate from a single source which implies homogeneity in active phytochemicals or it may arrive from different sources which makes it necessary to investigate the effectiveness of extracts of trees of various origins.

Globally, around 33% of the food produced is lost and not consumed. A high percentage of harvested fresh produce is wasted yearly by microbial spoilage^4,5^. Mango (*Mangifera indica*) is a tropical fruit rich with fibers, vitamin C, vitamin A, polyphenols, carotenoids, carbohydrates, and calcium. Various types of mangoes have wide ranges of nutritional facts^6^. India is the largest producer of mango in the world^7^. Mango availability in the market is affected by its susceptibility to postharvest diseases and to chill injuries. The short shelf life of mango increases its prices in markets that depend totally on imported fruit^8^. Mango is the second most important internationally traded tropical fruit straight after banana, which gives it a high economical value^9^. In 2020, imported mangoes costed Qatar 70.8MM QR making 8.12% of the total cost of imported fruits, this number highlights the significance of wastage of mango in storage for the country economy^10^.

During the storage time, the physiological changes that occurs on fruit make them more prone to the development of microorganisms^11^. Postharvest losses in mango can reach 50% in some developing countries such as Ethiopia^9^. The main fungal species that causes losses in mango are *Alternaria. alternata* and *Colletotrichum gloeosporioides*. Infection of mango by *A. alternata* can occur through the lenticels and cause rot mainly on the side of the fruit or through the ends of the stem to cause stems rot^12^. On the other hand, *C. gloeosporioides* is the causative agent of mango anthracnose, one of the most devastating and hard to control diseases in this fruit^13^.

A high percentage of the spoilage controllers currently used in the agricultural domain are either polluting or costly^14^. Chemical antifungals and antimicrobials used for spoilage control increase fruit and vegetables shelf-life, but they also leave their damaging fingerprints on the environment and pose, in many cases, potential risks on human health^15^. In addition, the controlled microorganisms are acquiring, with time, resistance to the commonly used antimicrobials, which is a problem that requires immediate attention^16,17^. This lead many scientists to explore plants and other sources of natural products to replace chemicals used in-fields and at post-harvest level^18^.

Within natural products, higher plants phytochemicals have valuable characteristics that help in curing several diseases^19^. Various researches have documented antifungal effectiveness of higher plants extracts at the level of spores germination and/or mycelium growth. Among the tested plants, *Prosopis species* in general and *P. juliflora* in particular have shown antioxidant, anti-inflammatory, antibacterial, antifungal, and anti-tumor activities^20^. The stability of active phytochemicals with time is one of the desired characteristics when exploring natural products^21^. *P. juliflora* leaf ethanolic extract has been proven by our team as an effective antimicrobial against a list of fungi and bacteria*.* The previous study showed also stability of the antimicrobial activity over time, for up to six months, which encouraged the team to explore the in vivo value of the extract^22^. This study aims to construct the phylogeny tree of *P. juliflora* in the municipality of Doha, Qatar, to evaluate genetic relatedness among different samples, variation of in vitro antifungal effectiveness among samples collected from different places was also evaluated. The study aims then to test *P. juliflora* water-soluble leaf ethanolic (PJ-WS-LE) extract curative and preventive effect against artificially inoculate *A. alternata* and *C. gloeosporioides* in mango. Moreover, It aims finally at evaluating the effect of the extract on its own and when embedded in chitosan, as a protective coating material for mango samples. Chitosan (poly β-(1–4)*N*-acetyl-d-glucosamine), used as stabilizer of our extract, is a non-toxic biodegradable polymer that has shown in previous studies an antifungal activity against many fungal types including *C. gloeosporioides*^23^. PJ-WS-LE extract is a solvent free solution that can be prepared using an economically feasible method, its antimicrobial activity results makes it a promising replacement of chemically synthesized fungicides. The extract has shown high efficacy in increasing strawberry and cucumber samples shelf-lives and in maintaining their quality storage parameters when used as coating material^24,25^.

## Materials and methods

### *Prosopis juliflora* leaves collection and processing for Ribotyping

**Figure 1:**
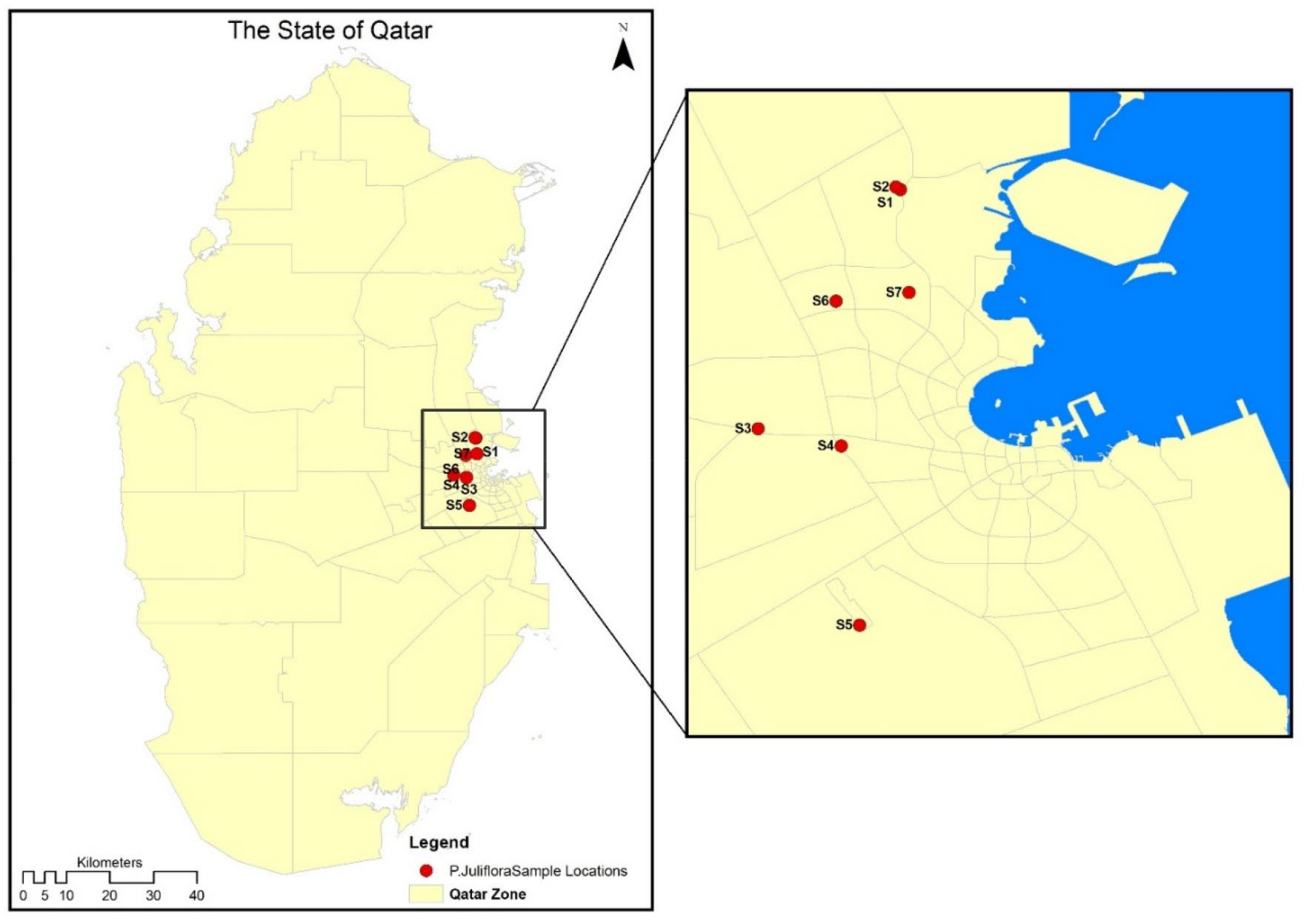
Location map of collection sites of *P. juliflora* leaf samples (ArcGIS software).

*Prosopis juliflora* species of the genus *Prosopis*, family of Fabaceae had its genetic variation in Doha evaluated. Seven samples of *P. juliflora* leaves were collected from six different locations in Doha, Qatar, during five field trips. Plant leaves were collected after proper permissions and all methods were carried out in accordance with relevant guidelines and regulations. Trees in all locations were naturally growing around urbanization areas in their normal arid habitat without artificial irrigation, samples were collected from fully mature trees. Table 1 shows the samples details. Figure 1 shows the location sites of where the samples were collected on the map of Qatar, Doha. Leaf samples were kept in sterile labeled bags until having reached the laboratory where few leaflets were washed with sterile distilled water and sterilized using 70% ethanol to be used for DNA extraction.

**Table 1:** Location details of the collection sites of *P. juliflora* leaves.

Sample name	Latitude	Longitude	Location	Sampling trip number
S1	25°22′24"N	51°29′48"E	Qatar University field	1
S2	25°22′27"N	51°29′43"E	Qatar University field	2
S3	25°17′50"N	51°27′05"E	Al Rayyan	2
S4	25°17′30"N	51°28′40"E	Al Amir street	3
S5	25°14′05"N	51°29′01"E	Abu Hamour	4
S6	25°20′16"N	51°28′34"E	Al Duhail	4
S7	25°20′26"N	51°29′58"E	Rawdat Al Faras	5

### Ribotyping analysis

The leaflets of each sample were transferred into a sterile mortar previously cooled at -20 ˚C and used for DNA extraction following the kit manufacturer instructions (DNeasy Plant Mini Kit-QIAGEN-USA).

Extracted DNA of each sample were subject to PCR using ITS1 and ITS4 primers. PCR products obtained were purified using the Invitrogen Quick PCR Purification Kit (QIAGEN, Germany) as indicated by the manufacturer and sequenced using Sanger sequencer (3130/3130xl DNA Analyzers, Thermofisher Scientific, USA) as previously described^22^.

Sanger sequencer raw data was read using BioEdit software. Basic Local Alignment Search Tool (BLAST) network services of the National Centre for Biotechnology Information (NCBI) database were used to compare the obtained sequences to the existing sequences. Sequences were submitted to NCBI for accession numbers. The various *P. juliflora* ribosomal sequences obtained were also uploaded on MEGA-X software and the phylogeny tree was generated using the neighbor-joining algorithm^26^.

### Minimum inhibitory concentrations of PJ-WS-LE extracts prepared using leaf samples collected from various locations against *A. alternata* and *C. gloeosporioides*

#### Preparation of PJ-WS-LE extract

Fresh, young full leaves of *P. juliflora* were collected from various locations as indicated in Fig. 1. Samples were washed, dried and ground into powder to be used in the preparation of PJ-WS-LE extract as previously described^22^. Briefly, every 20 g of the leaf powder were incubated in 200 mL of 70% ethanol for 48 h. The supernatant has its solvent evaporated, the extract was then re-dissolved in sterile distilled water. Only water-soluble phytochemicals were tested by centrifuging the final preparation tubes and excluding the pellet. Stock solution of 100 g L^−1^ was stored at 4 °C to be used for later experiments. PJ-WS-LE extract concentration used in treatments was 8 g L^−1^ which is double the highest minimum inhibitory concentration of the extract against spoiling microorganisms as previously determined^22^.

#### Determination of minimum inhibitory concentration

The MIC test was conducted in a sterile 96-well plate, with each well containing 100 μl of potato dextrose broth (PDB) (HIMEDIA-India). Every four wells made one replication, nine different concentrations of the crude extracts were tested (1:1 dilutions) ranging from 42 to 0.16 g L^−1^. Wells were then inoculated with one of the two tested microorganisms’ spore suspensions (*A. alternata* and *C. gloeosporioides*). The last three rows are control rows: no spores and no extract control wells, negative control with spores but no extract wells, and positive control with spores and 10 µl of the fungicidal Clatrimazole (1%) wells.

Fungal spore suspensions were adjusted to the range of 10^4^ spores L^−1^ using a 10 day old fungal plate and sterile distilled water, the spore concentration was calculated using a heamatocytometer.

Fungal growth in each well was monitored using Resazurin (HIMEDIA-India) dye. Upon cells division, Resazurin changes its color from blue to pink and fluorescent^27^. Results were taken within 48 h of incubation at 25 °C. MIC was recorded as the last extract concentration that shows no change in the color of Resazurin within the incubation period.

### Curative and preventive effects of PJ-WS-LE extract against *A. alternata* and *C. gloeosporioides* induced infection in mangoes

#### Pathogens

The two fungal strains used *C. gloeosporioides* and *A. alternata* were obtained from our laboratory collection, Department of Biological and Environmental Sciences, Qatar University, Qatar. Both fungal isolates were previously isolated from locally collected fruit samples. Isolates were molecularly identified by sequencing the Internal Transcribed Spacer (ITS) regions of fungal ribosomal DNA (rDNA) that was amplified by PCR. Identified fungal isolates were given the strains code of AaltQU17 for *A. alternata* and CgloQU17 for *C. gloeosporioides*^22^. Preserved cultures were sub-cultured on potato dextrose agar (PDA) plates and incubated at 25 °C for 10 days. Plates were then flooded with 10 mL of sterile distilled water each, to prepare the needed spores suspension solutions. The concentrations of spores suspensions were adjusted to 10^6^ spores L^−1^ using a heamatocytometer^18^.

#### Fruit

The mango (*Mangifera indica*) type known as Neelam imported from India was used in the experiments. Fruit were bought from the whole sale market upon their arrival to the country. Only undamaged mature fruit were used in the experiment. Fruits chosen were ripen but not yet soft with firmness average of 20 ± 5.1 N, weight average of 177.61 ± 0.2 g and TSS average of 70 ± 5.3%. Fruit were first washed with sink water and sterilized twice with 70% ethanol to be then washed with sterile distilled water and left to air dry.

#### Preventive and curative effects of PJ-WS-LE extract

Wounded mango fruit were used during the experiment, the wounds were made through three needle pricks (2 mm deep) in three different places for each plant using a sterile syringe. A completely randomized design was used and each treatment was made of a triplicate of 10 fruit each. The experiment was repeated twice.

PJ-WS-LE extract of leaves collected from Qatar university field was first tested for its efficacy in preventing fungal contamination in wounded mango fruit (preventive effect). Therefore, the wounded zone of each fruit was sprayed with 8 g L^−1^ PJ-WS-LE extract and then left to air-dry. Once dry the fruit were sprayed again with the extract at the same concentration and left to dry. Control fruit were only treated with sterile distilled water without the plants extract. After two hours all wounds were inoculated with 20 μL of conidia aqueous solution (10^6^ spores mL^−1^) of one of the tested fungi. The extract was then tested for its ability to cure fungal contamination in wounded fruit. Therefore, wounds were inoculated first with 20 μL of conidia aqueous solution (10^6^ spores mL^−1^) and left to dry. Wounds were then sprayed twice with 8 g L^−1^ PJ-WS-LE extract.

All mangoes were stored in sterilized plastic trays inside an incubator at 25 °C and 75% humidity. Fruit were observed every 24 h for 5 days for *C. gloeosporioides* inoculated fruit and for 10 days for *A. alternata* inoculated fruit. Three parameters were recorded at the end of the experiment: disease incidence (DI), disease severity (DS), and percent plant extract efficacy (%EE). To calculate disease severity, the diameter of the infected area of each fruit was measured in two perpendicular directions and mean diameter mycelial growth was calculated^28,29^.

$$\mathrm{DI}=\frac{(\mathrm{Number\, of\, rotten\, fruit})\times 100}{\mathrm{Total\, number\, of\, fruit}}$$

$$\mathrm{DS }=\frac{(\mathrm{Average\, lesion\, diameter\, of\, treated\, plants})\times 100}{\mathrm{Average\, Lesion\, diameter\, of\, control\, plants})}$$

$$\mathrm{\%EE}=\frac{(\mathrm{Disease\, incidence\, in\, Control\, batch}-\mathrm{Disease\, incidence\, in\, treated\, batch})\times 100}{\mathrm{Disease\, incidence\, in\, Control\, batch}}$$

#### End of the trial samples firmness

At the end of the trial, remaining mango fruit were tested for their flesh quality using a penetrometer (Agriculture Solutions, USA) to test the flesh firmness. Fruit were peeled, then the stainless steel probe of the instrument was inserted in three different points towards the equator of the fruit. Firmness in Newton was recorded and compared with standard fruit firmness to judge fruit quality^18^.

### Effectiveness of PJ-WS-LE extract as long-term coating material and the preservative value of its chitosan-embedded form

#### Coating solutions preparation

Chitosan solution of 1% concentration was prepared by stirring chitosan powder (CAS 9012-76-4, Himedia, India) in 1% glacial acetic acid (IsoLab, Germany) overnight. The final chitosan solution pH was adjusted to 5.6 using 0.1 M NAOH (Sigma-Aldrich, Germany). To prepare PJ-WS-LE extract chitosan-embedded coating material, filter-sterilized PJ-WS-LE extract stock solution was added to 1% chitosan to achieve a final concentration of 8 g L^−1^^30^.

#### Samples preparation

Eighty-four mango samples chosen as described above, were divided into four groups of 18 samples each. Samples were divided into four treatment batches and treated as following:

- Batch A: non-treated fruit.
- Batch B: PJ-WS-LE extract at 8 g L^–1^ was used to spray the fruit.
- Batch C: 1% chitosan was used to spray the fruit.
- Batch D: 8 g L^−1^ PJ-WS-LE extract embedded in 1% chitosan was used to spray the fruit.

Every experimental replicate was made up of three mango samples that were stored together in one sterile bag at 4 °C. The number of replications per treatment was six. The experiment was repeated twice^31^.

#### Evaluation of sensory quality

A five-points scale was used for the evaluation of the sensory quality of the samples for overall quality, smell, and color change. The attributes were evaluated weekly using the fruit of one experimental replicate. Scores were given using the following scale: 5 points indicate “extremely liked”, 4 points indicate “liked”, 3 points indicate “acceptable” 2 points indicate “disliked” and 1 point indicates “extremely disliked”. The weekly average score per batch was also calculated^32^.

#### Estimation of weight loss

Upon treatment at day zero, all mango samples were weighed and their weights were recorded as initial weights. Weights of all remaining samples were measured at the end of every week. The variation between the start weight and weekly weights is calculated as weekly weight loss. The average percent of weekly weight loss of each batch was calculated^32^.

#### Determination of samples firmness

The samples of each experimental replicate evaluated on a weekly basis had their firmness measured as previously described. The weekly average samples firmness (N) of every treatment batch was also calculated^33^.

#### pH measurement

Mango fruit of each experimental replicate were blended weekly into juice, after filtration, a digital pH meter (Jenway, UK) was used to measure pH. The weekly average fruit pH of every treatment batch was also calculated. The pH meter was calibrated using a buffer solution of pH 7^34^.

#### Total soluble solids (TSS) measurement

Total soluble solids of the prepared mango juice samples were measured in percent brix using a refractometer (ANTAHI, New Zealand). The weekly average fruit TSS (%) for each treatment batch was also calculated. The refractometers was calibrated using distilled water^35^.

#### DPPH radical scavenging assay

A 1/10 mango juice dilution was prepared using sterile distilled water. 100 μL of each dilution was mixed with 1 mL of 2,2-diphenyl-1-picrylhydrazyl (DPPH) (100 mg L^−1^) to be incubated in the dark at 37 °C for 45 min. After incubation, samples were centrifuged and the pellet was discarded. The intensity of the change in color of the supernatant was measured by spectrophotometry at 517 nm using methanol as a blank. 100 μL of methanol in 1 mL DPPH was used as the control for the experiment. Percent radical scavenging activity was calculated as per the below formula:

$$ \% {\text{ radical scavenging activity}}\, = \,\left( {{\text{absorbance of the control solution}} - {\text{ absorbance of the juice sample}}} \right)*{1}00/{\text{absorbance of the control solution}}. $$

The weekly average % radical scavenging activity for each treatment batch was finally calculated^31^.

### Statistical analysis

The experimental design used was Completely Randomized Design (CRD). One-way ANOVA followed by Tukey Post-Hoc test was used to evaluate the significance of the weekly percent change in weight among treatment batches at *P* ≤ 0.05. The significances of pH and TSS variation within different treatment batches were evaluated using One-way ANOVA test at *P* ≤ 0.05. Data was presented as average ± standard error of the Means (SEM). SPSS (Ver. 27, SPSS Inc. Chicago, USA) was used to perform the statistical analysis tests.

## Results

### DNA fingerprinting results

**Figure 2:**
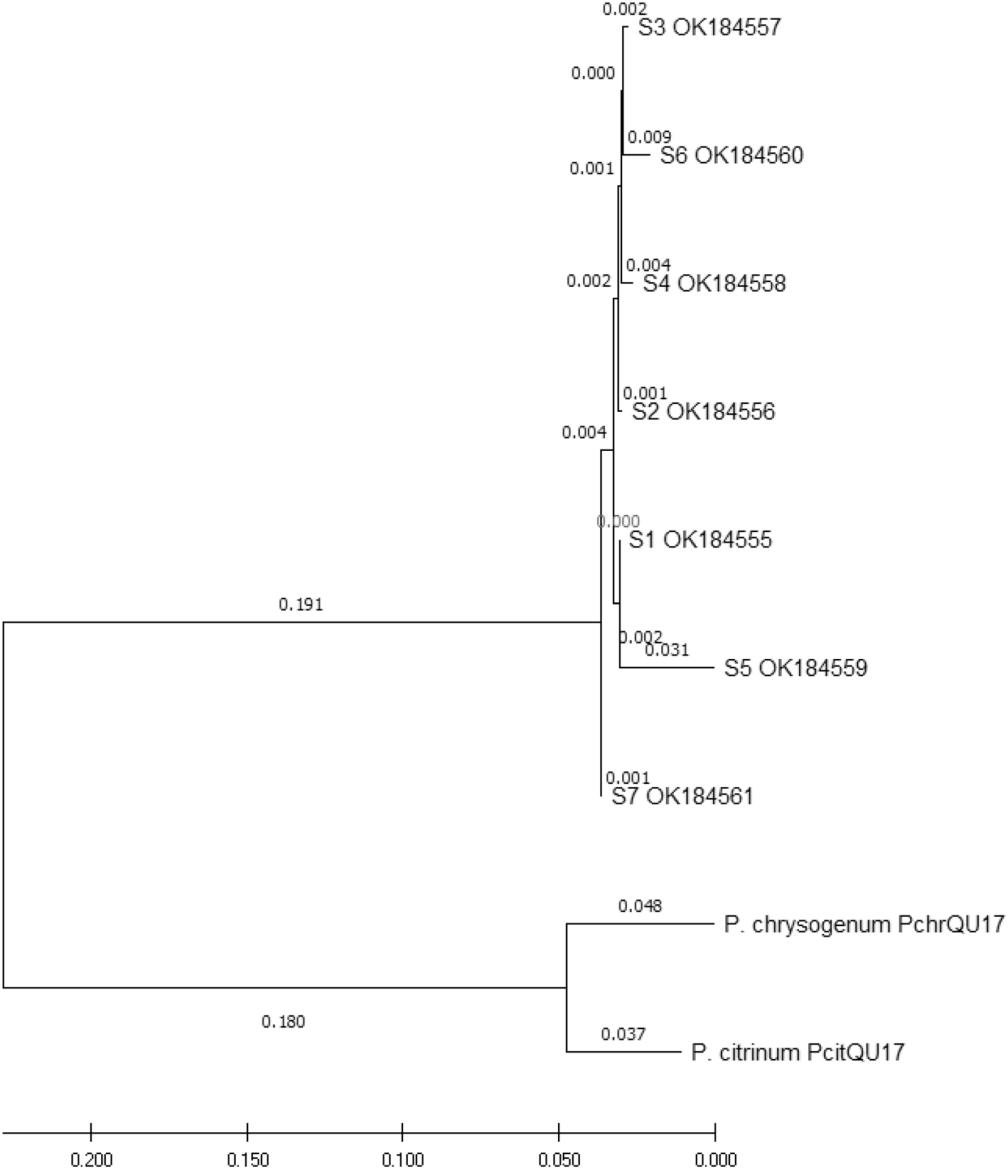
Phylogenetic analysis, using the neighbor-joining method, of *P. juliflora* based on the ITS1-5.8S-ITS2 sequences of seven isolates collected from different locations in Doha-Qatar. *Penicillium* sequences (*P. chrysogenum* and *P. citrinum*) are the outgroup.

Upon submission to NCBI, the seven *P. juliflora* rDNA sequences were given accession numbers (Table 2). The blasting of the sequences on NCBI, in order to compare them with the database, showed percent similarities between 96 and 99% to the sequence representing *P. juliflora* 18S ribosomal RNA gene, partial sequence; internal transcribed spacer 1, 5.8S ribosomal RNA gene, and internal transcribed spacer 2, complete sequence; and 28S ribosomal RNA gene, partial sequence with accession number JX139107.1 (originated from India). Neighbor joining phylogeny tree of the sequences shows very low variation among the *P. juliflora* isolates collected from different places in Doha (Fig. 2).

**Table 2:** Similarity results of the PCR products blasting.

Sample name	Sequence length (bp)	Accession number	Percentage of nucleotides identity (%) to sequence number JX139107.1
S1	932	OK184555	98.84
S2	877	OK184556	98.46
S3	532	OK184557	97.90
S4	535	OK184558	98.84
S5	533	OK184559	95.91
S6	732	OK184560	97.87
S7	723	OK184561	98.65

### MICs evaluation of crude extract prepared using *P. juliflora* leaves samples from various locations against *A. alternata* and *C. gloeosporioides*

**Figure 3:**
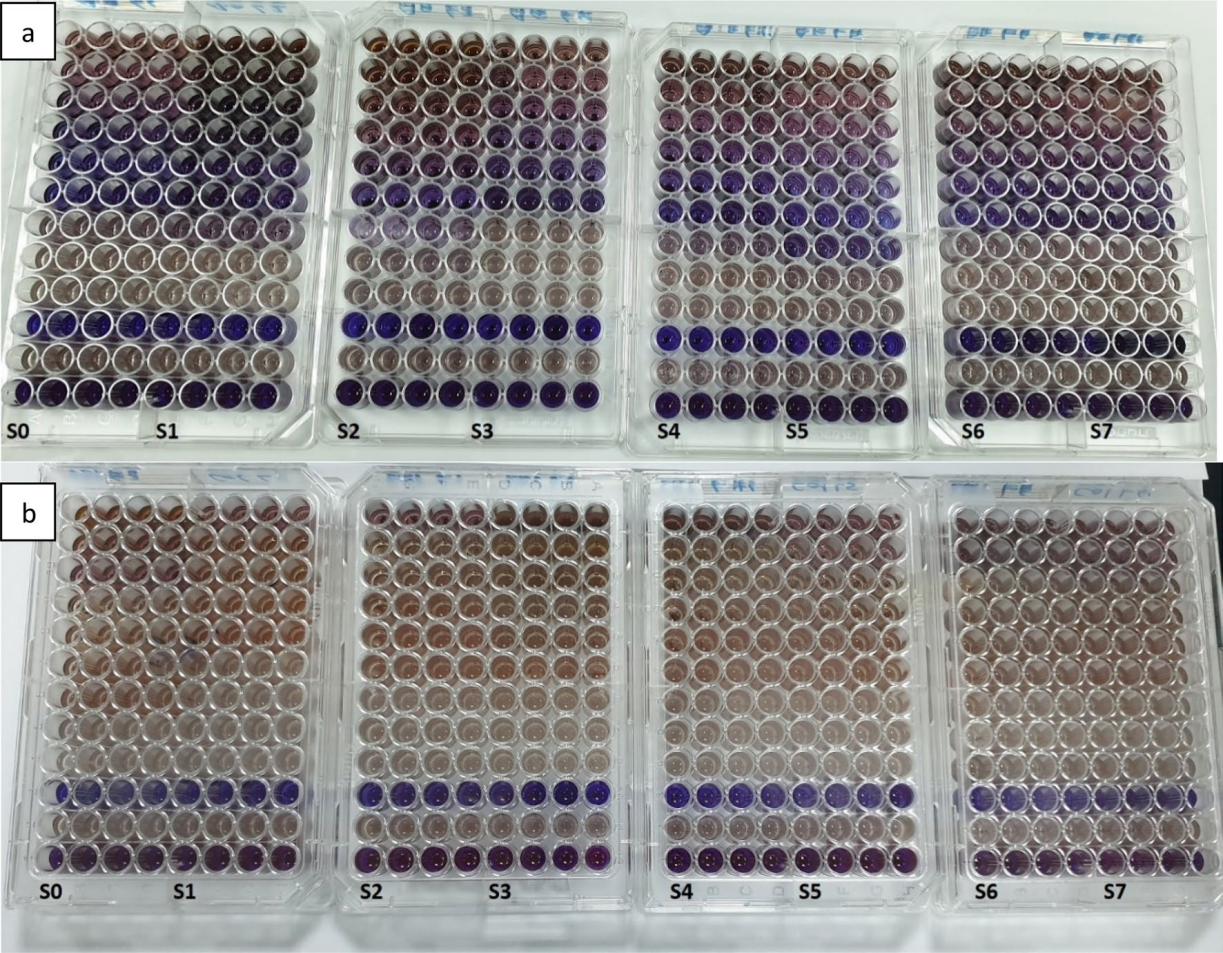
MICs of PJ-WS-LE extracts prepared using *P. juliflora* leaves collected from different locations in Qatar against *A. alternata* (**a**) and *C. gloeosporioides* (**b**). S0: old extract from the laboratory. S1 to S7: extract prepared using leaves of sample S1 to S7. Rows 1 to 9 contains the following concentrations of PJ-WS-LE extract in g L^−1^: 42, 21, 10.5, 5.2, 2.6, 1.3, 0.6, 0.3 and 0.16. Row 10 contains no spores, row 11 contains no extract and row 12 contains 1% Clatrimazole.

The MICs of the extracts prepared using leaves of trees from different locations showed homogeneity in their results. The slight one raw variation shown in the case *A. alternata* is due to the difficulty in preparing extracts of exactly the same concentration at every run, which in turn is due to the difficulty in controlling how much of the extract is water-soluble. The MICs of the extract against *A. alternata* are within the range obtained previously in our laboratory, which is 1 g L^−1^ (Fig. 3). As previously described, PJ-WS-LE extract did not show a total growth inhibition of *C. gloeosporioides*. However, the change in color in the 96-well plate experiment shows a dose-dependent effectiveness in lowering fungal growth (Fig. 3).

### Curative and preventive effects of PJ-WS-LE extract against *A. alternata* and *C. gloeosporioides* induced infection in mangoes

**Figure 4:**
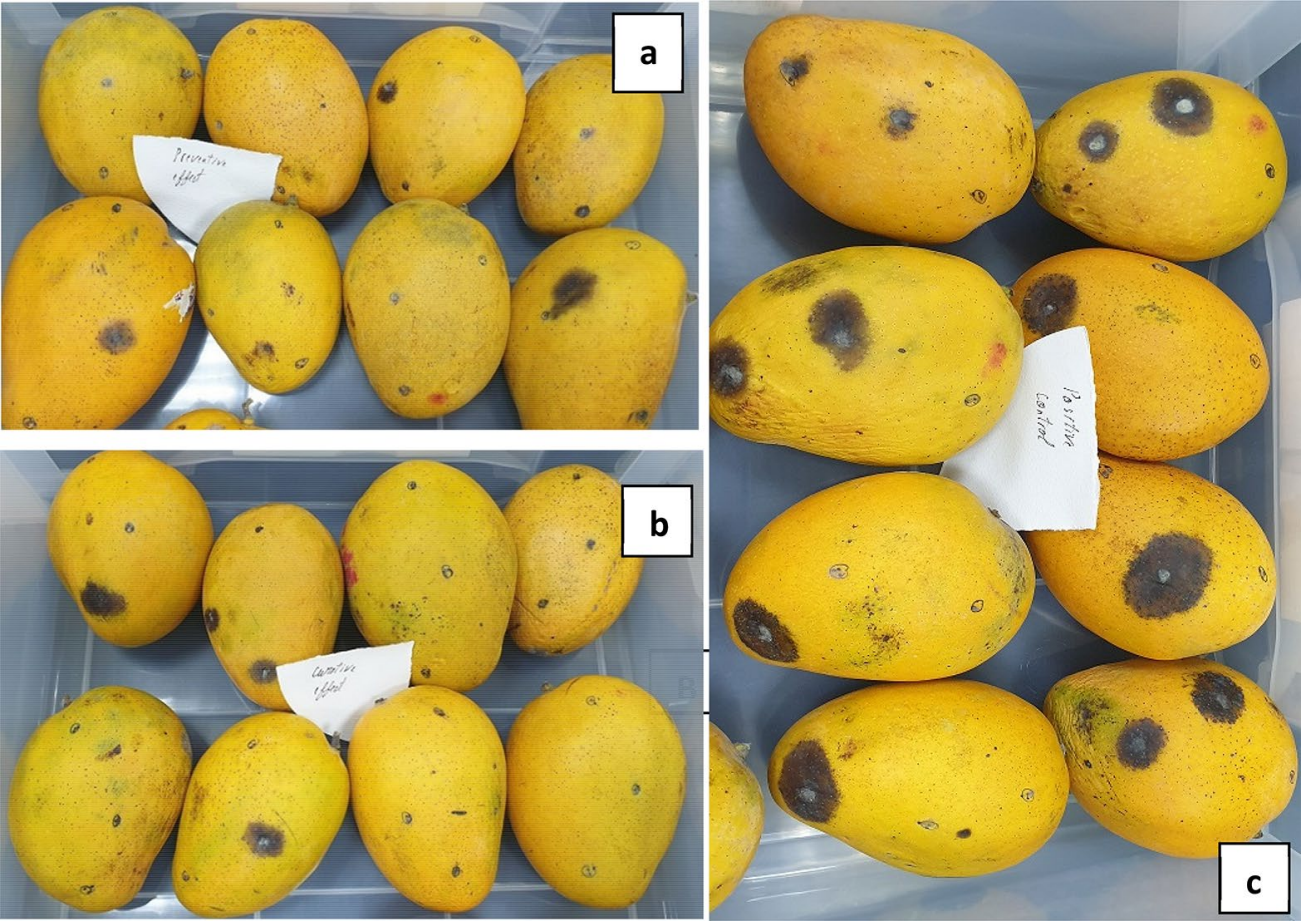
Disease incidence in mango samples inoculated with *A. alternata*. All samples are 10 days old after treatment at 25 °C. (**a**) Preventive effect of PJ-WS-LE extract. (**b**) Curative effect of PJ-WS-LE extract. (**c**) Control samples inoculated with *A. alternata* and not treated with PJ-WS-LE extracts.

**Figure 5:**
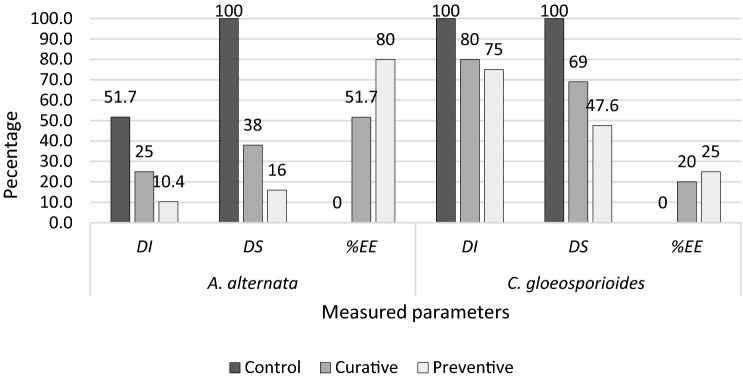
Curative and preventive measured parameters of PJ-WS-LE extract against *A. alternata* and *C. gloeosporioides* inoculated in mango samples. Disease incidence (DI), disease severity (DS), and percent plant extract efficacy (%EE).

In the case of *A. alternata* artificially inoculated mango fruit, PJ-WS-LE extract showed high efficacy in reducing infection. The incidence rate went down from 51.7% in control samples to 10.3% in treated samples with preventive treatment and to 25% in treated samples with curative treatment (Fig. 4). PJ-WS-LE extract at 8 g L^−1^ had an efficacy of 80% against *A. alternata* infection by the end of the 10 d storage period. Disease severity was also reduced in treated samples. Samples with preventive treatment (average lesion diameter = 3.7 mm) that showed infection demonstrated a disease severity of 16% when compared to untreated control samples (average lesion diameter = 11.5 mm) (Fig. 5). The effect of PJ-WS-LE extract on mango firmness was evaluated at day 10. ANOVA test showed a significant average firmness conservation (*P* = 0.00 ≤ 0.01) between plants exposed to the extract (curative and preventive treatment) (14.93 N) and the untreated plants (7.4 N).

The efficacy of PJ-WS-LE extract in reducing *C. gloeosporioides* incidence on artificially inoculated fruit was low, preventive treatment showed a disease incidence rate reduction of 25% (extract efficacy). Although extract efficacy in controlling mango anthracnose does not account for total eradication, PJ-WS-LE extract exhibited significant reduction in disease severity, which was reduced by more than 50%, from a control average lesion diameter of 14.7 mm to a preventive treatment average lesion diameter of 7.0 mm by the end of the 5 d storage period (Fig. 5). The effect of PJ-WS-LE extract on mango firmness was also evaluated at day 5. One-way ANOVA test showed a significant average firmness conservation (P = 0.00 ≤ 0.01) in samples exposed to the extract (curative and preventive treatments) (26.74 N) compared to the control samples firmness average (12.62 N).

### PJ-WS-LE extract long-term efficacy as coating material and the preservative value of its chitosan-embedded form

#### Sensory evaluation

**Figure 6:**
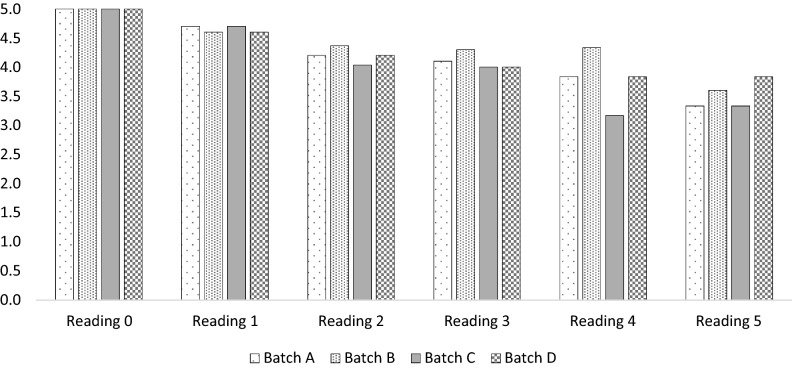
Weekly sensory evaluation of mango fruit exposed to four different treatments (**A** negative control non-treated fruit; **B** PJ-WS-LE extract at 8 g L^−1^ was used to treat the fruit; **C** 1% chitosan was used to treat the fruit; **D** 8 g L^−1^ PJ-WS-LE extract embedded in 1% chitosan was used to treat the fruit) stored at 4 °C for 5 weeks.

The weekly changes of mangoes sensory characteristics stored at 4 °C were evaluated. Figure 6 shows the sensory scores changes of the fruit of each of the treatment batches.

The overall mangoes’ sensory scores declined with storage time. Nevertheless, samples of treatment batches B and D coated with 8 g L^−1^ of PJ-WS-LE extract individually and embedded in 1% chitosan conserved better sensory scores during the five week storage time.

Score from 1 to 5 are: 5 points indicate “extremely liked”, 4 points indicate “liked”, 3 points indicate “acceptable” 2 points indicate “disliked” and 1 point indicates “extremely disliked”.

### Weight loss

**Figure 7:**
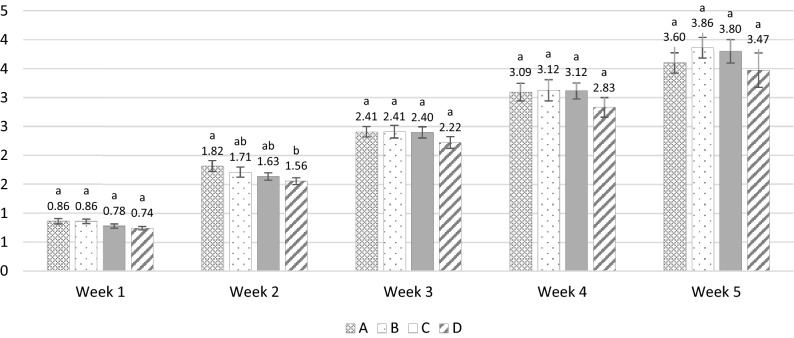
Weekly average percentage change in weight of the overall mangoes exposed to various treatments ± SE (**A** negative control non-treated fruit; **B** PJ-WS-LE extract at 8 g L^−1^ was used to treat the fruit; **C** 1% chitosan was used to treat the fruit; **D** 8 g L^−1^ PJ-WS-LE extract embedded in 1% chitosan was used to treat the fruit). ^ab^Treatment columns marked with different letters have their percent change in weight values significantly different as per the results of the one-way ANOVA Post-Hoc Tukey test for weekly data.

The weekly percent change in weight of every treatment batch was calculated (Fig. 7). Weight loss increased with time, nevertheless, PJ-WS-LE extract embedded in chitosan showed efficacy in reducing weight decline in the fruits.

A significant increase of percent change of weight of mango samples was observed in all treatment categories with the passage of storage time. Mangoes coated with 8 g L^−1^ of PJ-WS-LE extract in 1% chitosan had the lowest percent change in weight with an observable weekly increase. One-way ANOVA test showed a significant effect of treatment type on percent weight change in the overall data of all weeks, post-Hoc Tukey test shows that treatment D (8 g L^−1^ PJ-WS-LE extract embedded in 1% chitosan) lead significantly to lower percent weight loss compared to the control fruit (batch A) in the overall data (*p* = 0.012 ≤ 0.05).

### Weekly changes of physical and chemical properties (firmness, pH, TTS, antioxidant activity, and ascorbic acid level)

Mango samples treated differently had their weekly physical parameters monitored. Table 3 shows the weekly results of averages firmness, pH and total soluble solids.

**Table 3:** Average firmness, pH, TSS and DPPH radical scavenging activity ± SE of mango samples exposed to different treatments (A: negative control non-treated fruit; B: PJ-WS-LE extract at 8 g L^−1^ was used to treat the fruit; C: 1% chitosan was used to treat the fruit; D: 8 g L^−1^ PJ-WS-LE extract embedded in 1% chitosan was used to treat the fruit) throughout 5 weeks.

Treatment batch	Storage week	Firmness (N)	pH	TSS (%)	DPPH RSA (%)
Batch A negative control samples	1	20.39 ± 7.23	4.71 ± 0.053	71.3 ± 6.33	36.22 ± 6.9
	2	23.80 ± 3.18	4.51 ± 0.051	69.0 ± 0.00	37.27 ± 0.5
	3	12.14 ± 1.02	4.84 ± 0.111	69.3 ± 6.88	62.28 ± 1.5
	4	17.66 ± 5.44	4.71 ± 0.063	68.3 ± 3.38	64.71 ± 7.1
	5	9.98 ± 0.80	4.90 ± 0.090	70.7 ± 3.71	-
Batch B coated with 8 mg/ml PJ-WS-LE extract	1	11.41 ± 2.67	4.82 ± 0.055	77.0 ± 0.57	43.47 ± 0.4
	2	17.58 ± 7.52	4.80 ± 0.072	72.7 ± 1.67	34.54 ± 3.1
	3	12.58 ± 0.88	4.87 ± 0.082	63.7 ± 2.33	68.21 ± 4.0
	4	12.3 ± 1.12	4.94 ± 0.117	73.0 ± 3.05	70.97 ± 3.8
	5	7.26 ± 0.83	5.19 ± 0.113	76.7 ± 3.75	-
Batch C coated with 1% chitosan	1	18.07 ± 4.47	4.67 ± 0.034	74.9 ± 2.02	54.33 ± 7.4
	2	7.43 ± 2.21	4.92 ± 0.119	69.7 ± 2.40	37.88 ± 2.7
	3	10.18 ± 2.37	4.99 ± 0.013	70.7 ± 2.18	44.65 ± 3.2
	4	13.72 ± 2.08	4.74 ± 0.052	75.3 ± 3.71	74.82 ± 6.0
	5	14.49 ± 3.73	5.02 ± 0.043	77.3 ± 8.96	-
Batch D coated with 8 mg/ml PJ-WS-LE extract in 1% chitosan	1	20.91 ± 10.1	4.69 ± 0.020	73.3 ± 3.33	58.04 ± 3.7
	2	15.33 ± 2.15	4.71 ± 0.022	69.7 ± 0.88	73.44 ± 5.1
	3	19.07 ± 1.40	4.65 ± 0.119	75.7 ± 9.92	55.29 ± 4.0
	4	23.45 ± 6.37	4.26 ± 0.083	81.7 ± 1.66	80.95 ± 3.6
	5	21.43 ± 5.22	4.31 ± 0.172	86.7 ± 3.33	-

Mango samples of batches A, B and C showed a decrease in their firmness during the storage time although samples of batch D showed a kind of firmness stability. The results of the mango juice pH of all treatment batches increased weekly, except for batch D which samples showed pH levels decrease. When all of the weeks’ pH results for each batch were combined and tested using one-way ANOVA followed by Tukey test, the overall results showed significant differences in pH levels of mango juices of different batches (P = 0.00 ≤ 0.01). Tukey test shows that batch D samples had significantly lower pH than the other samples (average pH  4.51).

Total soluble solids results showed fluctuation in TSS levels throughout the five weeks of storage. One-way ANOVA test, followed by Tukey test done on the overall results show a significant difference in the average TSS levels (P = 0.009 ≤ 0.01). Tukey test results show that samples of batch D had significantly higher TSS levels when compared to others with an average TSS level of 81.7% compared to an average TSS level of 69.4% in the case of the control samples (batch A).

DPPH radical scavenging activity of mango samples of different treatment batches was measured for four weeks. The antioxidant activity increased with time in all mango samples. The highest increase was observed in mango samples coated with chitosan and PJ-WS-LE extract (batch D) followed by samples coated with chitosan alone (batch C).

## Discussion

Sequence number JX139107.1 has originated from India. Knowing that *P. juliflora* is not a native species of Qatar, the similarity between the indicated sequence and our sequences could mean that workers of Indian origin may have brought the first seeds of the plant to the country, hence it is resistant to similar climatic conditions and due to its pharmaceutical importance among Indian society^20^. The low variation in climate among the different places in Qatar does not allow for genetic variation among the different isolates. Considering the possibility that all the trees in the country could originate from the same country (India) explains also the similarity among the sequences. A previous study on the diversity within and among the populations of *P. cineraria* and *P. juliflora* collected from different locations in Qatar was conducted on 12 samples, six of each species. Genetic variation was explored using Inter Simple Sequence Repeat (ISSR) and Random Amplified Polymorphic DNA (RAPD) markers. The analysis divided the twelve genotypes into two distinct clusters by species and showed genetic variations among samples of *P. juliflora*, however, the variation was inconsistent between the two methods used^36^. Collections of more samples and the usage of other genetic markers are needed to confirm genetic identity of *P. juliflora* in Qatar.

The results of MICs are consistent with the low genetic variation shown in the phylogeny analysis of *P. juliflora* in Qatar. The homogeneity in climate and in soil type in the collection areas, as well as the age of the tree that is relatively new to the country, support the obtained results and imply that the types of active phytochemicals are similar in all trees. Therefore, if to be utilized, all *P. juliflora* trees in Qatar would have the same antimicrobial effectiveness. The lack of variation in the in vitro results has led to the usage of PJ-WS-LE extract of a single source in the in vivo assays.

The MIC of PJ-WS-LE extract against *A. alternata* was shown to be around 1 g L^−1^ (1001 ppm) which is high compared to the concentration of chemical fungicides used in-field to control mango anthracnose. Systemic fungicides are usually used at concentration between 25 and 50 ppm, examples include Carbendazim, Propiconazole and others. Non-systemic fungicide are usually used at higher concentrations that might reach 1000 ppm, which is very close to our PJ-WS-LE extract MIC, examples include Mancozeb, Copper oxychloride and others^37^. Nevertheless, PJ-WS-LE crude extract MIC cannot be compared to the concentrations of chemical fungicides as the extract is a mixture of active and non-active phytochemicals. In addition, PJ-WS-LE extract might need to be used at higher concentration, yet it has the advantage of being new, which means that spoiling fungi haven not yet developed resistant to it.

The protective effect of PJ-WS-LE extract against anthracnose caused by *C. gloeosporioides* in artificially inoculated mango samples was 25%. Similar anthracnose incidence inhibition rate was seen in inoculated avocadoes samples treated with *Aloe vera* extract. However, when *Aloe vera* extract was mixed with 1% chitosan, the disease incidence rate went down from 90% in the control samples to 20% in treated samples, similarly to our results, preventive treatment was more effective when it comes to *C. gloeosporioides*. Based on the results of the long term experiment involving chitosan, it is likely to have a higher extract efficacy against *C. gloeosporioides* when mixing PJ-WS-LE extract to chitosan^18^. As for the disease severity, PJ-WS-LE extract demonstrated a high success rate in lowering anthracnose lesion diameter by more than half with average lesion diameter of 7 mm in the preventive treatment samples. The result is somehow comparable to the final lesion diameter of treated avocado samples (diameter = 8.94 mm) as shown by Bill et al. Another study conducted on mangoes showed significant efficacy of *P. juliflora* leaf extracts in lowering anthracnose severity in inoculated samples. A comparison between the two extraction methods should be considered to improve our extract efficacy, however, it is vital to first examine the kind of solvent used by Deressa and Jalata, since it may have toxic antifungal activity all on its own^9^. A study conducted on Papaya samples, showed the effectiveness of different plant extracts, collected from Ambo and Haramaya, Ethiopia, in lowering anthracnose severity without totally inhibiting the fungal growth on plants, which indicates the difficulty in controlling mango anthracnose to a 100% level. In vivo experiments on mangoes showed much higher efficacy of PJ-WS-LE extract in protecting artificially inoculated samples from *A. alternata* (80%). Those results are in harmony with previous in vitro results showing total inhibition of *A. alternata* by PJ-WS-LE extract, while *C. gloeosporioides* showed only a decrease in mycelium growth^38^. One of the most common fungi causing mango postharvest diseases in the world is *A. alternata*, it can cause rot on the sides and stem ends, resulting in significant postharvest losses^39^. Controlling *A. alternata* using a naturally produced extract would add a valuable, safe and effective product to the agricultural world.

The experiment of long-term fruit preservation was conducted on mango samples stored at 4 °C and divided into four treatment batches. Samples coated with 8 g L^−1^ of PJ-WS-LE extract embedded in 1% chitosan (batch D) conserved sensory scores between acceptable and liked quality during the five week storage period, which highlighted the coating efficacy.

During the postharvest storage period, water evaporation leads to fruit shriveling and weight loss^40^. Mangoes coated with 1% chitosan and 8 g L^−1^ of the PJ-WS-LE extract showed the lowest percent change in weight with an observable weekly increase. At week 2, samples belonging to batch D showed significantly lower weight loss than samples of the control batch A. PJ-WS-LE extract increases water holding capacity when mixed with the edible coating of 1% chitosan. A recent study conducted on mangoes evaluated the effect of *Aloe Vera* gel embedded in 1% chitosan (CTS + AVG). Coated samples showed, similarly to our results, lower weight loss with time^8^.

Loss of firmness of fruit occurs during ripening by the activation of polygalacturonases (PG) and pectin methylesterase (PME) enzymes, which leads to the degradation of the middle lamella between parenchyma cells, loss of cellular turgidity, and cell wall disruption^41^. Samples firmness was evaluated with time, and all batches samples showed decrease in firmness with time except for the samples of batch D, which maintained their hardness during the five weeks of the experiment, thus giving a value to the coating material. Similarly, mango samples coated with (CTS + AVG) were significantly firmer than control samples^8^. The pH results of mango juices of different batches showed an increase in numbers throughout the storage weeks, except of batch D samples which had significantly lower pH values, thus indicating that different ripening chemical reactions are occurring inside the coated samples. Coating material also led to higher soluble sugar levels in mango juice samples, which might have played a role in quality maintenance. Tukey test results showed that samples of batch D have significantly higher TSS levels when compared to others. Finally, mango coating increased the DPPH radical scavenging activity. The highest increase was seen with mango samples of the coated batch D followed by samples of batch C. Similarly, (CTS + AVG) coated mango fruit showed higher antioxidant activity when compared to control samples^8^.

## Conclusion

PJ-WS-LE extract with its novel extraction and preparation method has been described in earlier studies of the team. The crude extract has shown a strong antimicrobial activity, stability with time, fungicidal activity against *A. alternata* and no phytotoxicity when applied on fruit. All these previous results opened doors to discover further PJ-WS-LE extract in vivo applications. The efficacy of the extract against *A. alternata* in mangoes and the significant decrease of mango diseases severity, add a strong value to PJ-WS-LE extract as a future replacement of chemical fungicides both for in-field and for postharvest applications. PJ-WS-LE extract embedded in edible coating has also been demonstrated as an effective coating material that can maintain mangoes quality at low temperature for up to five week storage. An investigation on the genetic variation of *P. juliflora* in Qatar, shows similar in vitro effectiveness of leaves collected from various *P. juliflora* trees in the country and proved too little or no genetic variation among samples, in addition to a common origin. Future studies will involve more fruit and vegetable, and more pathogenic spoiling agents to get to determine all the possible roles of this natural biological controller in agriculture.

## Data Availability

The datasets generated during and/or analyzed during the current study are available from the corresponding author on reasonable request.
